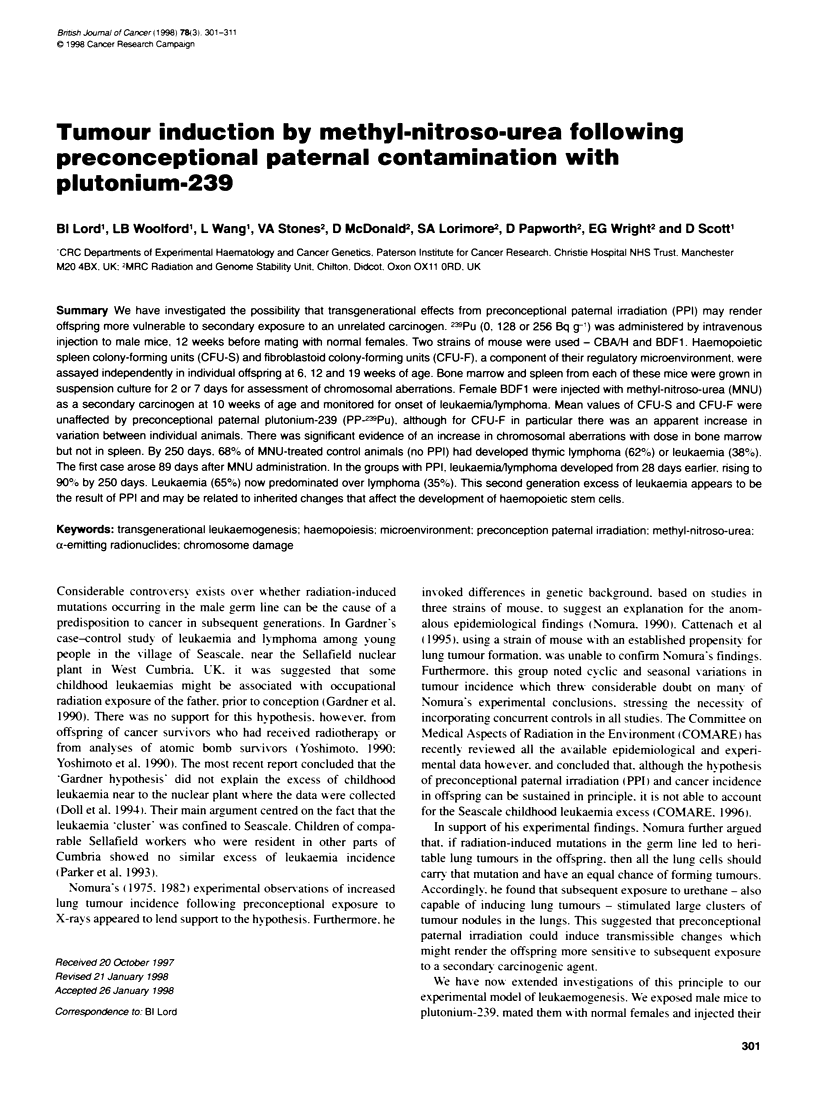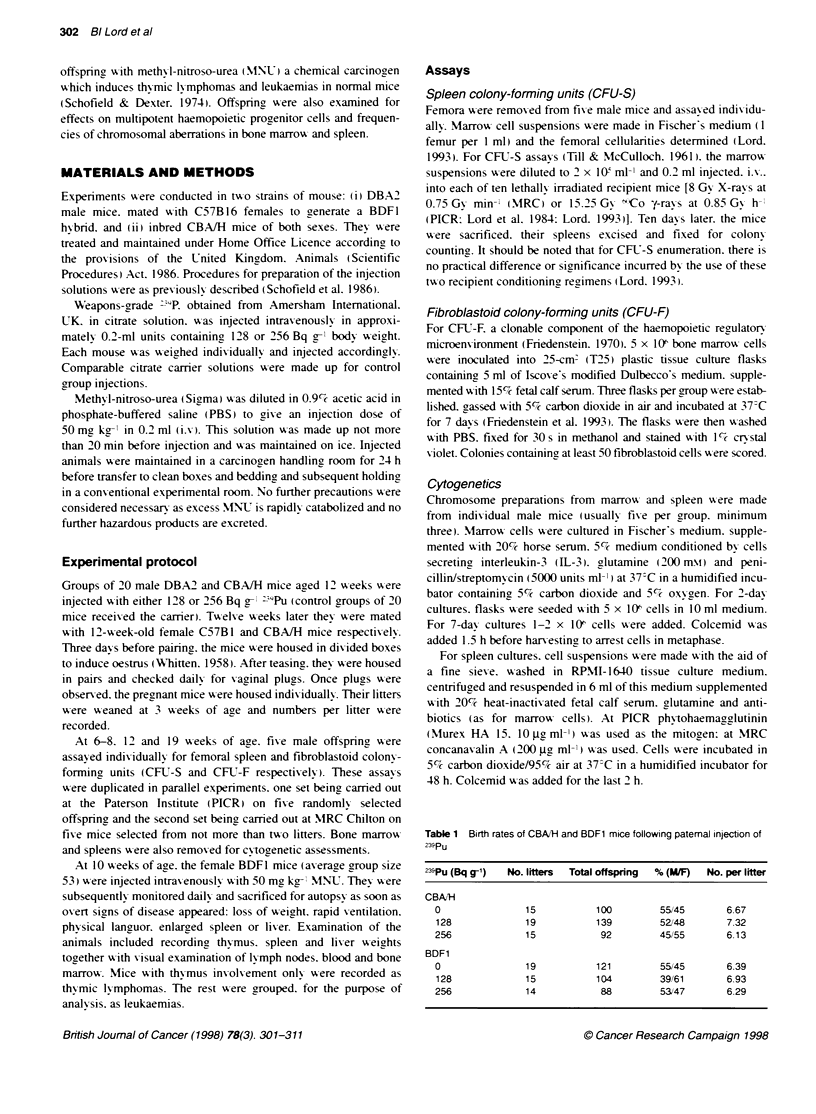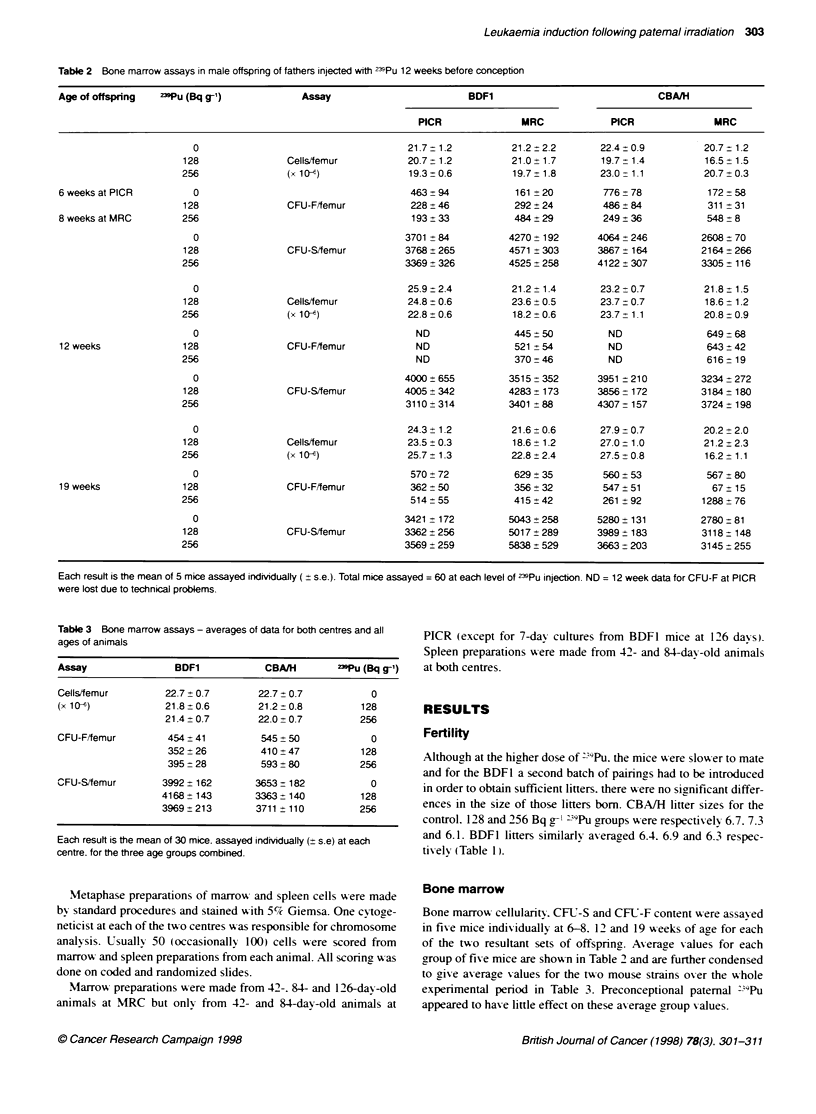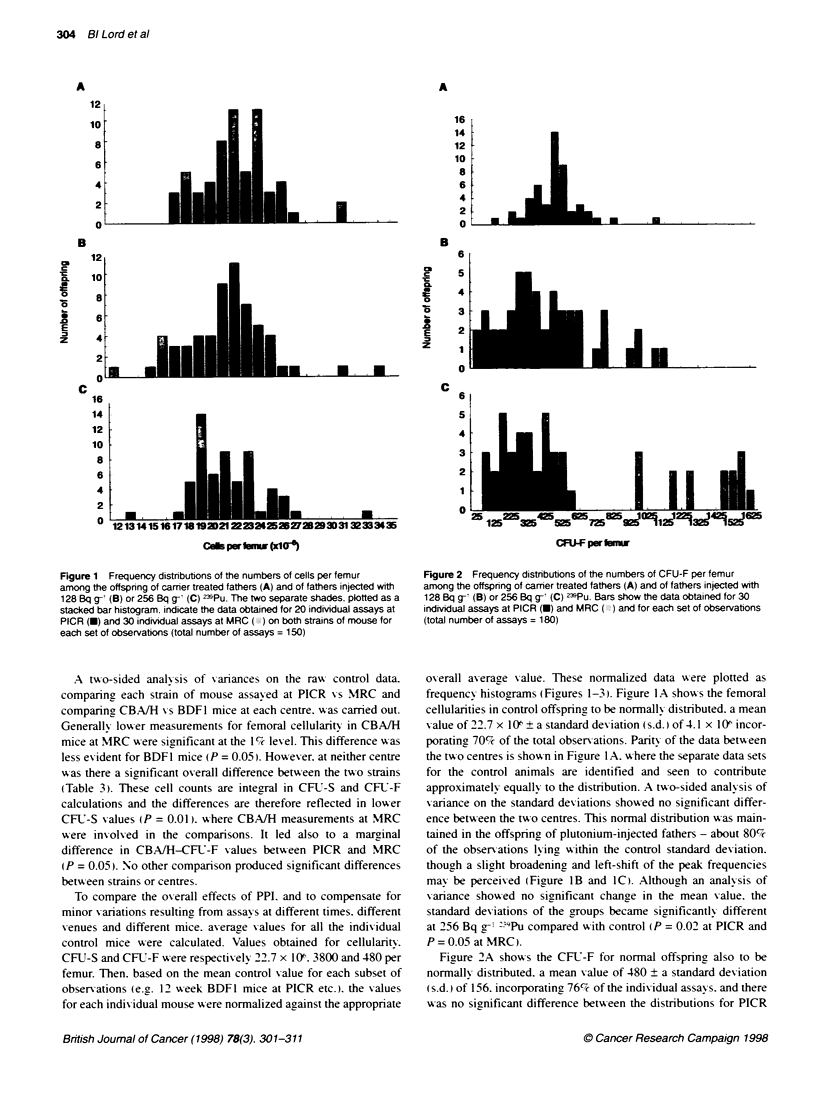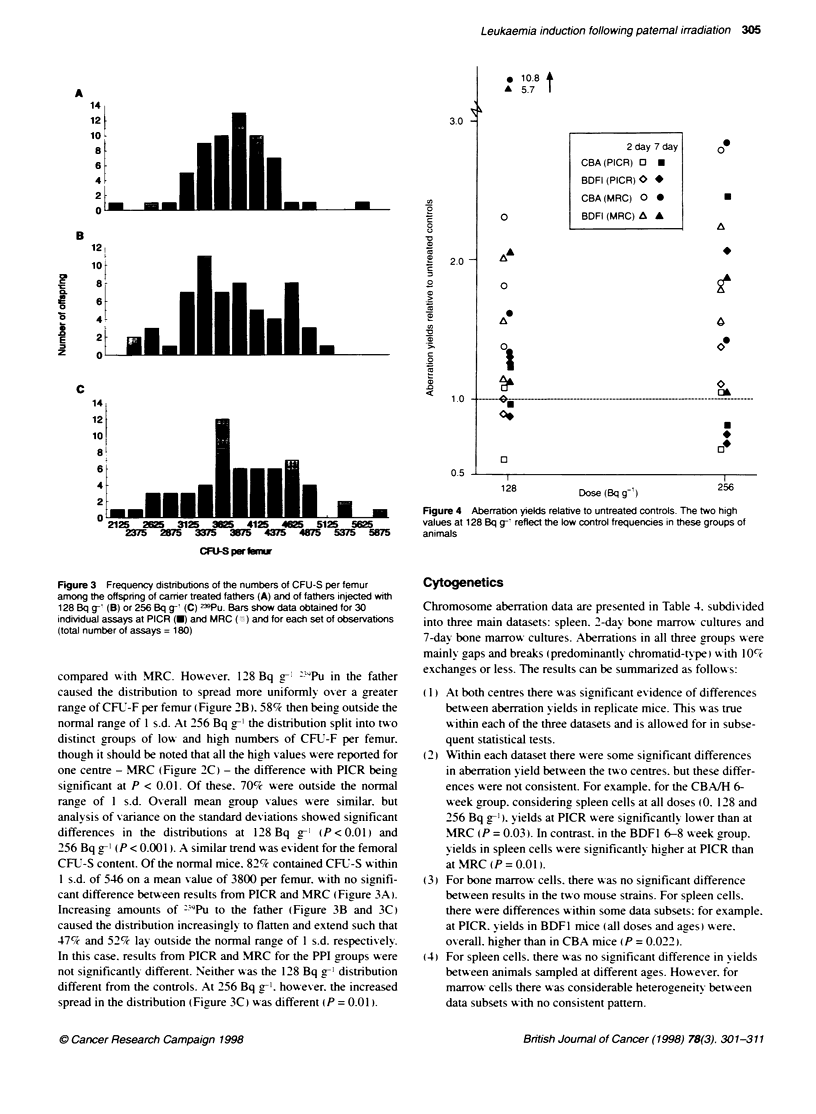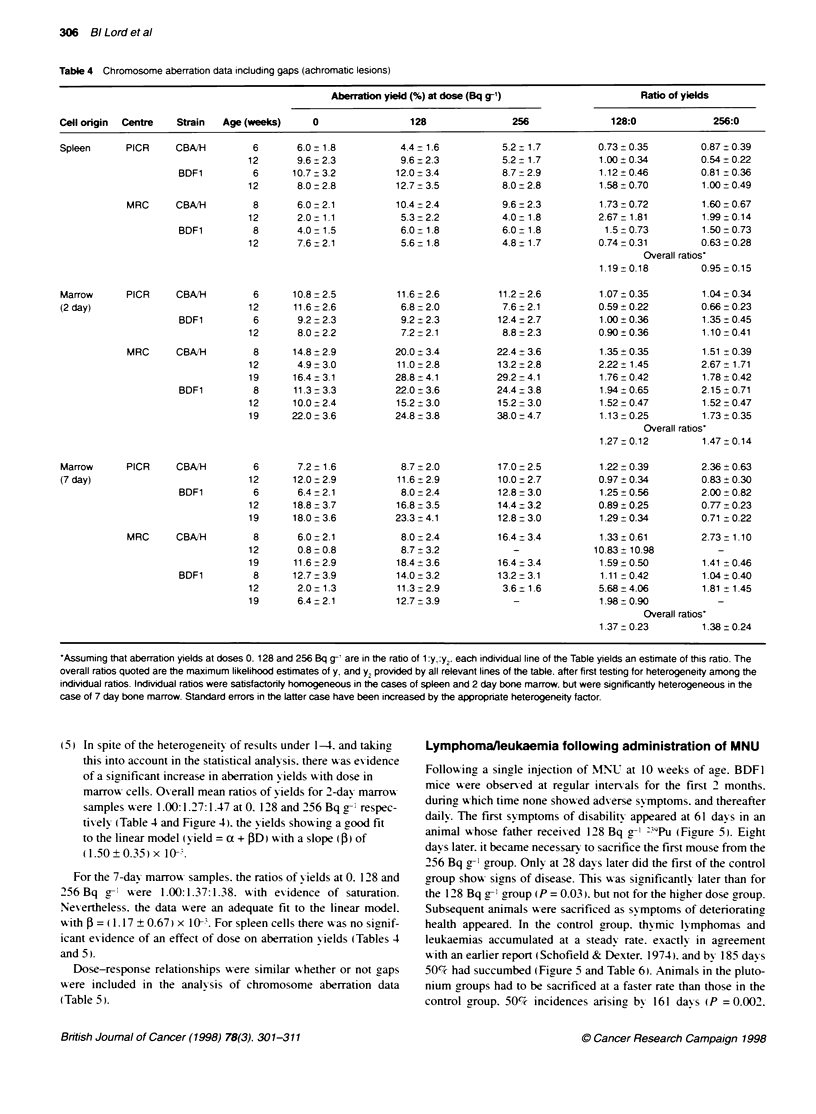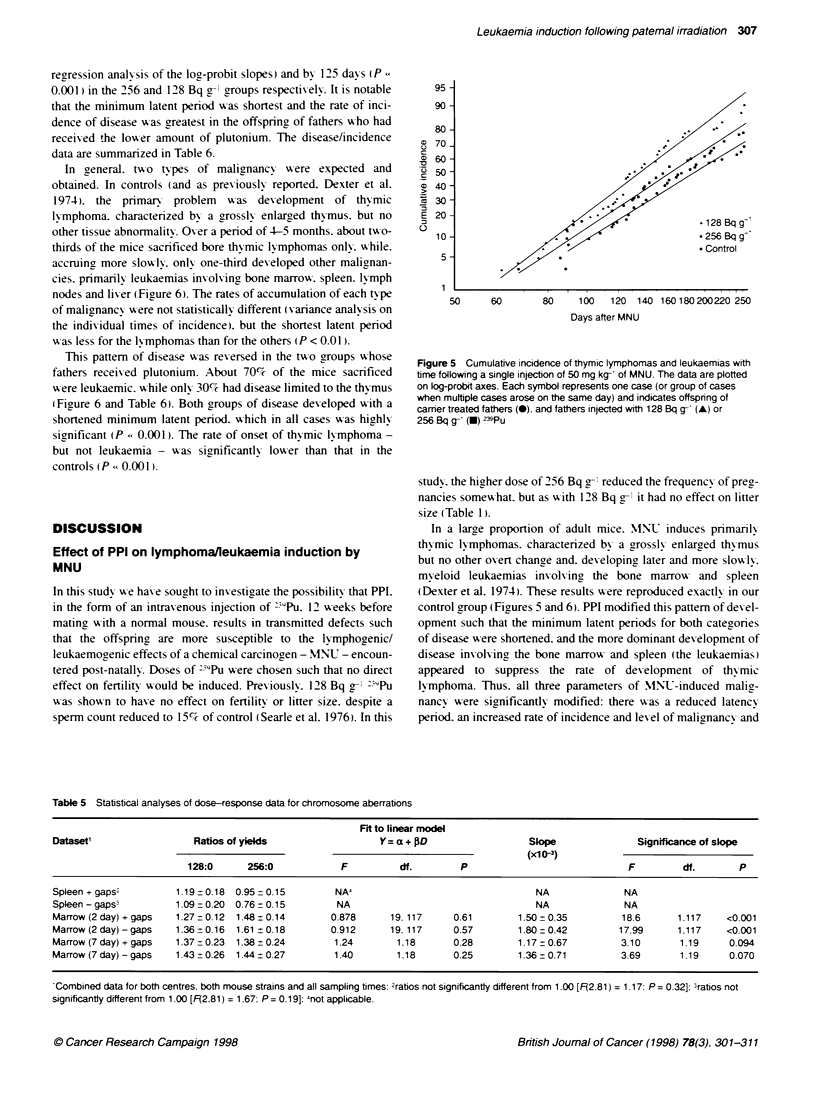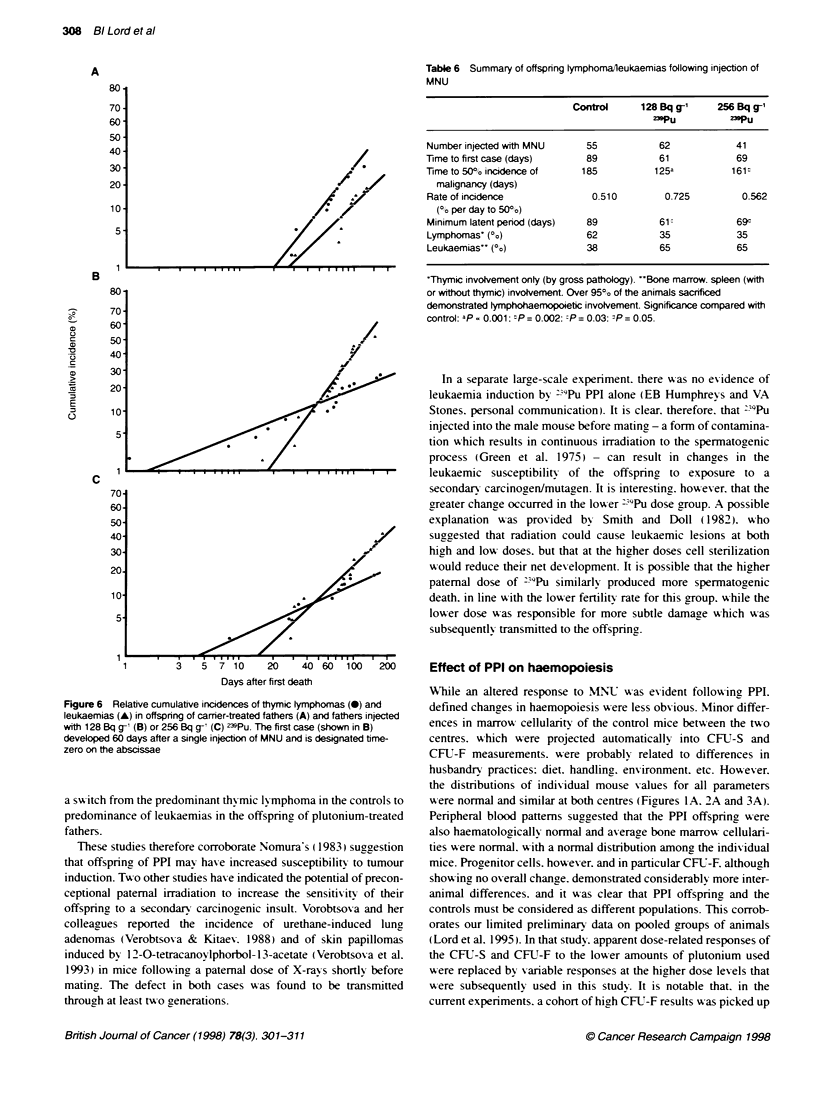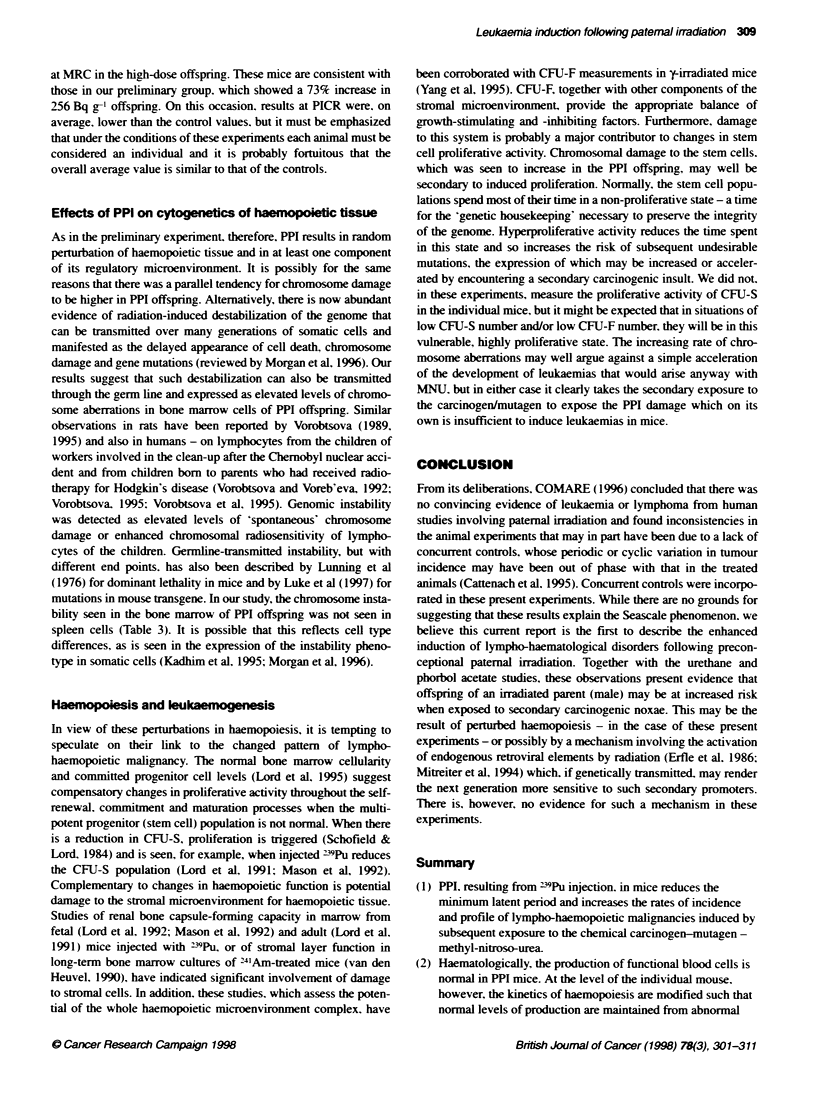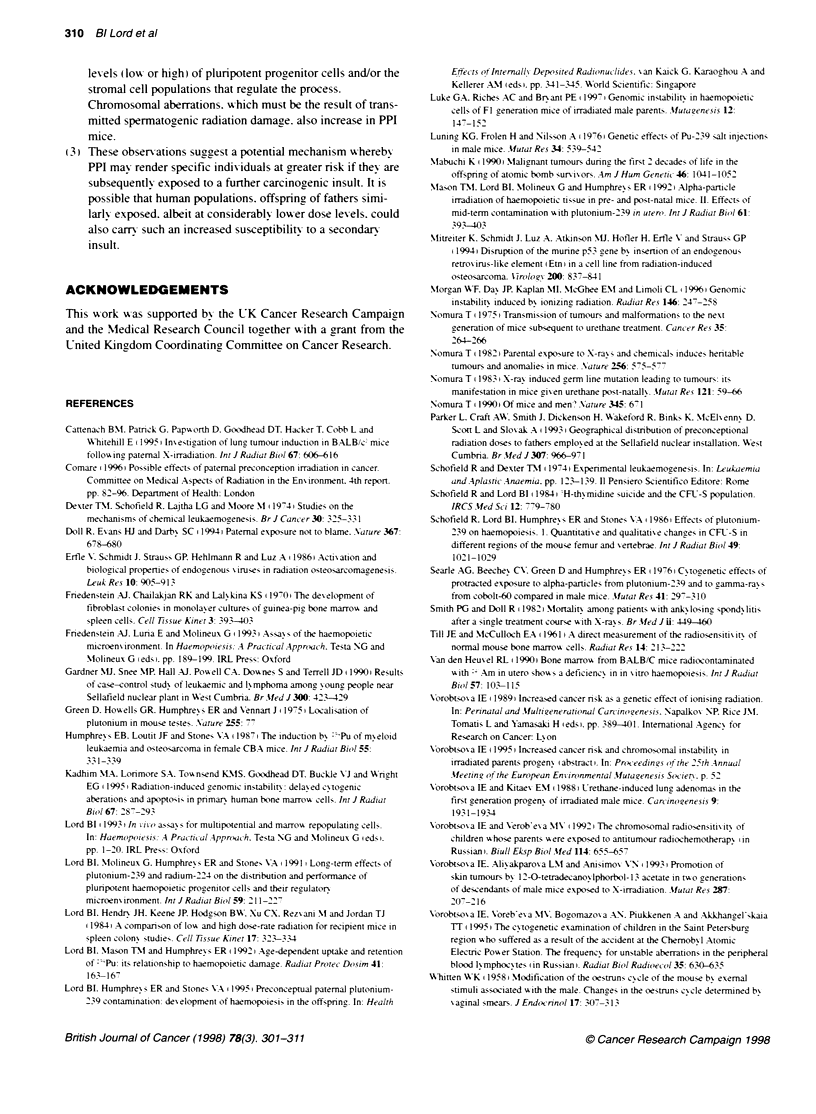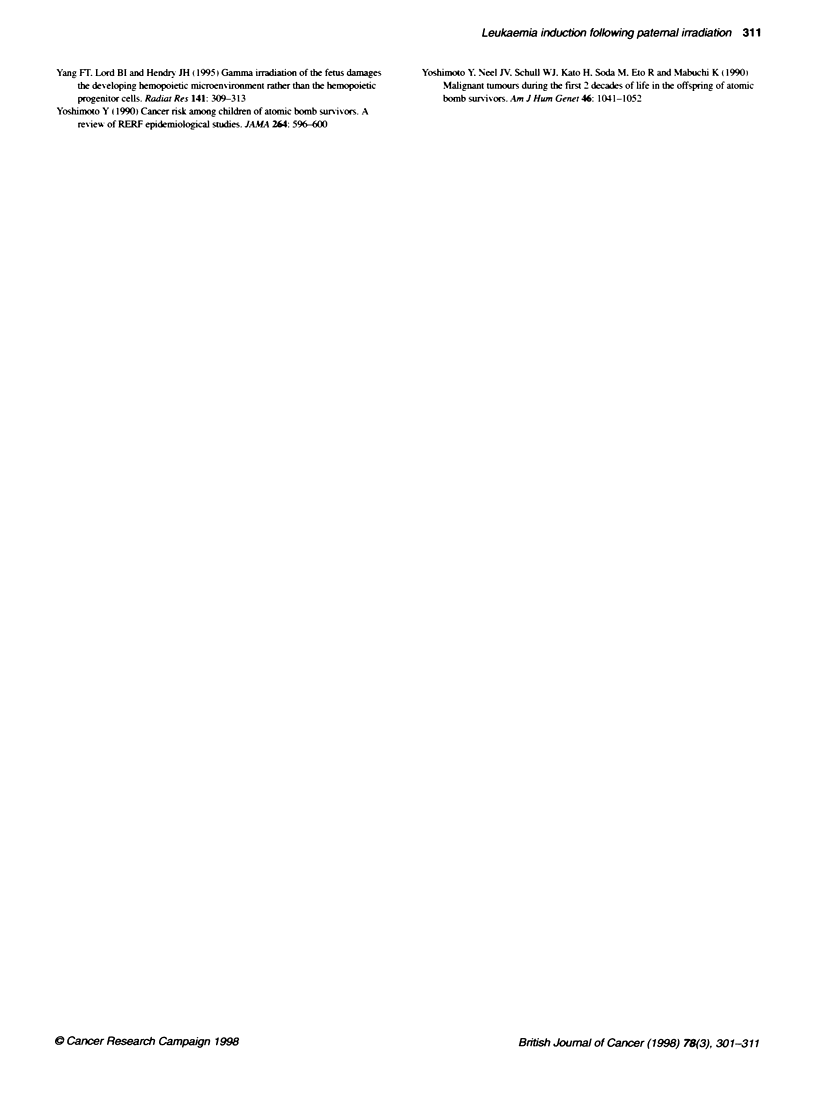# Tumour induction by methyl-nitroso-urea following preconceptional paternal contamination with plutonium-239.

**DOI:** 10.1038/bjc.1998.491

**Published:** 1998-08

**Authors:** B. I. Lord, L. B. Woolford, L. Wang, V. A. Stones, D. McDonald, S. A. Lorimore, D. Papworth, E. G. Wright, D. Scott

**Affiliations:** CRC Department of Experimental Haematology, Paterson Institute for Cancer Research, Christie Hospital NHS Trust, Manchester, UK.

## Abstract

**Images:**


British Journal of Cancer (1998) 78(3), 301–311
© 1998 Cancer Research Campaign

### Tumour induction by methyl-nitroso-urea following preconceptional paternal contamination with plutonium-239

BI Lord[1], LB Woolford[1], L Wang[1], VA Stones[2], D McDonald[2], SA Lorimore[2], D Papworth[2], EG Wright[2] and D Scott[1]

[1]CRC Departments of Experimental Haematology and Cancer Genetics, Paterson Institute for Cancer Research, Christie Hospital NHS Trust, Manchester M20 4BX, UK; [2]MRC Radiation and Genome Stability Unit, Chilton, Didcot, Oxon OX11 0RD, UK

**Summary** We have investigated the possibility that transgenerational effects from preconceptional paternal irradiation (PPI) may render offspring more vulnerable to secondary exposure to an unrelated carcinogen. $^{239}$Pu (0, 128 or 256 Bq g$^{-1}$) was administered by intravenous injection to male mice, 12 weeks before mating with normal females. Two strains of mouse were used – CBA/H and BDF1. Haemopoietic spleen colony-forming units (CFU-S) and fibroblastoid colony-forming units (CFU-F), a component of their regulatory microenvironment, were assayed independently in individual offspring at 6, 12 and 19 weeks of age. Bone marrow and spleen from each of these mice were grown in suspension culture for 2 or 7 days for assessment of chromosomal aberrations. Female BDF1 were injected with methyl-nitroso-urea (MNU) as a secondary carcinogen at 10 weeks of age and monitored for onset of leukaemia/lymphoma. Mean values of CFU-S and CFU-F were unaffected by preconceptional paternal plutonium-239 (PP-$^{239}$Pu), although for CFU-F in particular there was an apparent increase in variation between individual animals. There was significant evidence of an increase in chromosomal aberrations with dose in bone marrow but not in spleen. By 250 days, 68% of MNU-treated control animals (no PPI) had developed thymic lymphoma (62%) or leukaemia (38%). The first case arose 89 days after MNU administration. In the groups with PPI, leukaemia/lymphoma developed from 28 days earlier, rising to 90% by 250 days. Leukaemia (65%) now predominated over lymphoma (35%). This second generation excess of leukaemia appears to be the result of PPI and may be related to inherited changes that affect the development of haemopoietic stem cells.

**Keywords:** transgenerational leukaemogenesis; haemopoiesis; microenvironment; preconception paternal irradiation; methyl-nitroso-urea; α-emitting radionuclides; chromosome damage

Considerable controversy exists over whether radiation-induced mutations occurring in the male germ line can be the cause of a predisposition to cancer in subsequent generations. In Gardner's case–control study of leukaemia and lymphoma among young people in the village of Seascale, near the Sellafield nuclear plant in West Cumbria, UK, it was suggested that some childhood leukaemias might be associated with occupational radiation exposure of the father, prior to conception (Gardner et al, 1990). There was no support for this hypothesis, however, from offspring of cancer survivors who had received radiotherapy or from analyses of atomic bomb survivors (Yoshimoto, 1990; Yoshimoto et al, 1990). The most recent report concluded that the 'Gardner hypothesis' did not explain the excess of childhood leukaemia near to the nuclear plant where the data were collected (Doll et al, 1994). Their main argument centred on the fact that the leukaemia 'cluster' was confined to Seascale. Children of comparable Sellafield workers who were resident in other parts of Cumbria showed no similar excess of leukaemia incidence (Parker et al, 1993).

Nomura's (1975, 1982) experimental observations of increased lung tumour incidence following preconceptional exposure to X-rays appeared to lend support to the hypothesis. Furthermore, he invoked differences in genetic background, based on studies in three strains of mouse, to suggest an explanation for the anomalous epidemiological findings (Nomura, 1990). Cattenach et al (1995), using a strain of mouse with an established propensity for lung tumour formation, was unable to confirm Nomura's findings. Furthermore, this group noted cyclic and seasonal variations in tumour incidence which threw considerable doubt on many of Nomura's experimental conclusions, stressing the necessity of incorporating concurrent controls in all studies. The Committee on Medical Aspects of Radiation in the Environment (COMARE) has recently reviewed all the available epidemiological and experimental data however, and concluded that, although the hypothesis of preconceptional paternal irradiation (PPI) and cancer incidence in offspring can be sustained in principle, it is not able to account for the Seascale childhood leukaemia excess (COMARE, 1996).

In support of his experimental findings, Nomura further argued that, if radiation-induced mutations in the germ line led to heritable lung tumours in the offspring, then all the lung cells should carry that mutation and have an equal chance of forming tumours. Accordingly, he found that subsequent exposure to urethane – also capable of inducing lung tumours – stimulated large clusters of tumour nodules in the lungs. This suggested that preconceptional paternal irradiation could induce transmissible changes which might render the offspring more sensitive to subsequent exposure to a secondary carcinogenic agent.

We have now extended investigations of this principle to our experimental model of leukaemogenesis. We exposed male mice to plutonium-239, mated them with normal females and injected their

*Received 20 October 1997*
*Revised 21 January 1998*
*Accepted 26 January 1998*

*Correspondence to:* BI Lord offspring with methyl-nitroso-urea (MNU) a chemical carcinogen which induces thymic lymphomas and leukaemias in normal mice (Schofield & Dexter, 1974). Offspring were also examined for effects on multipotent haemopoietic progenitor cells and frequencies of chromosomal aberrations in bone marrow and spleen.

### MATERIALS AND METHODS

Experiments were conducted in two strains of mouse: (i) DBA2 male mice, mated with C57B16 females to generate a BDF1 hybrid, and (ii) inbred CBA/H mice of both sexes. They were treated and maintained under Home Office Licence according to the provisions of the United Kingdom, Animals (Scientific Procedures) Act, 1986. Procedures for preparation of the injection solutions were as previously described (Schofield et al, 1986).

Weapons-grade $^{239}$P, obtained from Amersham International, UK, in citrate solution, was injected intravenously in approximately 0.2-ml units containing 128 or 256 Bq $g^{-1}$ body weight. Each mouse was weighed individually and injected accordingly. Comparable citrate carrier solutions were made up for control group injections.

Methyl-nitroso-urea (Sigma) was diluted in 0.9% acetic acid in phosphate-buffered saline (PBS) to give an injection dose of 50 mg $kg^{-1}$ in 0.2 ml (i.v). This solution was made up not more than 20 min before injection and was maintained on ice. Injected animals were maintained in a carcinogen handling room for 24 h before transfer to clean boxes and bedding and subsequent holding in a conventional experimental room. No further precautions were considered necessary as excess MNU is rapidly catabolized and no further hazardous products are excreted.

### Experimental protocol

Groups of 20 male DBA2 and CBA/H mice aged 12 weeks were injected with either 128 or 256 Bq $g^{-1}$ $^{239}$Pu (control groups of 20 mice received the carrier). Twelve weeks later they were mated with 12-week-old female C57B1 and CBA/H mice respectively. Three days before pairing, the mice were housed in divided boxes to induce oestrus (Whitten, 1958). After teasing, they were housed in pairs and checked daily for vaginal plugs. Once plugs were observed, the pregnant mice were housed individually. Their litters were weaned at 3 weeks of age and numbers per litter were recorded.

At 6–8, 12 and 19 weeks of age, five male offspring were assayed individually for femoral spleen and fibroblastoid colony-forming units (CFU-S and CFU-F respectively). These assays were duplicated in parallel experiments, one set being carried out at the Paterson Institute (PICR) on five randomly selected offspring and the second set being carried out at MRC Chilton on five mice selected from not more than two litters. Bone marrow and spleens were also removed for cytogenetic assessments.

At 10 weeks of age, the female BDF1 mice (average group size 53) were injected intravenously with 50 mg $kg^{-1}$ MNU. They were subsequently monitored daily and sacrificed for autopsy as soon as overt signs of disease appeared: loss of weight, rapid ventilation, physical languor, enlarged spleen or liver. Examination of the animals included recording thymus, spleen and liver weights together with visual examination of lymph nodes, blood and bone marrow. Mice with thymus involvement only were recorded as thymic lymphomas. The rest were grouped, for the purpose of analysis, as leukaemias.

### Assays

#### *Spleen colony-forming units (CFU-S)*

Femora were removed from five male mice and assayed individually. Marrow cell suspensions were made in Fischer's medium (1 femur per 1 ml) and the femoral cellularities determined (Lord, 1993). For CFU-S assays (Till & McCulloch, 1961), the marrow suspensions were diluted to $2 \times 10^5$ $ml^{-1}$ and 0.2 ml injected, i.v., into each of ten lethally irradiated recipient mice [8 Gy X-rays at 0.75 Gy $min^{-1}$ (MRC) or 15.25 Gy $^{60}$Co $\gamma$-rays at 0.85 Gy $h^{-1}$ (PICR: Lord et al, 1984; Lord, 1993)]. Ten days later, the mice were sacrificed, their spleens excised and fixed for colony counting. It should be noted that for CFU-S enumeration, there is no practical difference or significance incurred by the use of these two recipient conditioning regimens (Lord, 1993).

#### *Fibroblastoid colony-forming units (CFU-F)*

For CFU-F, a clonable component of the haemopoietic regulatory microenvironment (Friedenstein, 1970), $5 \times 10^6$ bone marrow cells were inoculated into 25-$cm^2$ (T25) plastic tissue culture flasks containing 5 ml of Iscove's modified Dulbecco's medium, supplemented with 15% fetal calf serum. Three flasks per group were established, gassed with 5% carbon dioxide in air and incubated at 37°C for 7 days (Friedenstein et al, 1993). The flasks were then washed with PBS, fixed for 30 s in methanol and stained with 1% crystal violet. Colonies containing at least 50 fibroblastoid cells were scored.

#### *Cytogenetics*

Chromosome preparations from marrow and spleen were made from individual male mice (usually five per group, minimum three). Marrow cells were cultured in Fischer's medium, supplemented with 20% horse serum, 5% medium conditioned by cells secreting interleukin-3 (IL-3), glutamine (200 mM) and penicillin/streptomycin (5000 units $ml^{-1}$) at 37°C in a humidified incubator containing 5% carbon dioxide and 5% oxygen. For 2-day cultures, flasks were seeded with $5 \times 10^6$ cells in 10 ml medium. For 7-day cultures 1–2 $\times 10^6$ cells were added. Colcemid was added 1.5 h before harvesting to arrest cells in metaphase.

For spleen cultures, cell suspensions were made with the aid of a fine sieve, washed in RPMI-1640 tissue culture medium, centrifuged and resuspended in 6 ml of this medium supplemented with 20% heat-inactivated fetal calf serum, glutamine and antibiotics (as for marrow cells). At PICR phytohaemagglutinin (Murex HA 15, 10 µg $ml^{-1}$) was used as the mitogen; at MRC concanavalin A (200 µg $ml^{-1}$) was used. Cells were incubated in 5% carbon dioxide/95% air at 37°C in a humidified incubator for 48 h. Colcemid was added for the last 2 h.

**Table 1** Birth rates of CBA/H and BDF1 mice following paternal injection of $^{239}$Pu

| $^{239}$Pu (Bq $g^{-1}$) | No. litters | Total offspring | % (M/F) | No. per litter |
|---|---|---|---|---|
| CBA/H | | | | |
| 0 | 15 | 100 | 55/45 | 6.67 |
| 128 | 19 | 139 | 52/48 | 7.32 |
| 256 | 15 | 92 | 45/55 | 6.13 |
| BDF1 | | | | |
| 0 | 19 | 121 | 55/45 | 6.39 |
| 128 | 15 | 104 | 39/61 | 6.93 |
| 256 | 14 | 88 | 53/47 | 6.29 |

 © Cancer Research Campaign 1998

**Table 2** Bone marrow assays in male offspring of fathers injected with $^{239}$Pu 12 weeks before conception

| Age of offspring | $^{239}$Pu (Bq $g^{-1}$) | Assay | BDF1 | | CBA/H | |
|---|---|---|---|---|---|---|
| | | | PICR | MRC | PICR | MRC |
| | 0 | | 21.7 ± 1.2 | 21.2 ± 2.2 | 22.4 ± 0.9 | 20.7 ± 1.2 |
| | 128 | Cells/femur | 20.7 ± 1.2 | 21.0 ± 1.7 | 19.7 ± 1.4 | 16.5 ± 1.5 |
| | 256 | (× $10^{-6}$) | 19.3 ± 0.6 | 19.7 ± 1.8 | 23.0 ± 1.1 | 20.7 ± 0.3 |
| 6 weeks at PICR | 0 | | 463 ± 94 | 161 ± 20 | 776 ± 78 | 172 ± 58 |
| | 128 | CFU-F/femur | 228 ± 46 | 292 ± 24 | 486 ± 84 | 311 ± 31 |
| 8 weeks at MRC | 256 | | 193 ± 33 | 484 ± 29 | 249 ± 36 | 548 ± 8 |
| | 0 | | 3701 ± 84 | 4270 ± 192 | 4064 ± 246 | 2608 ± 70 |
| | 128 | CFU-S/femur | 3768 ± 265 | 4571 ± 303 | 3867 ± 164 | 2164 ± 266 |
| | 256 | | 3369 ± 326 | 4525 ± 258 | 4122 ± 307 | 3305 ± 116 |
| | 0 | | 25.9 ± 2.4 | 21.2 ± 1.4 | 23.2 ± 0.7 | 21.8 ± 1.5 |
| | 128 | Cells/femur | 24.8 ± 0.6 | 23.6 ± 0.5 | 23.7 ± 0.7 | 18.6 ± 1.2 |
| | 256 | (× $10^{-6}$) | 22.8 ± 0.6 | 18.2 ± 0.6 | 23.7 ± 1.1 | 20.8 ± 0.9 |
| | 0 | | ND | 445 ± 50 | ND | 649 ± 68 |
| 12 weeks | 128 | CFU-F/femur | ND | 521 ± 54 | ND | 643 ± 42 |
| | 256 | | ND | 370 ± 46 | ND | 616 ± 19 |
| | 0 | | 4000 ± 655 | 3515 ± 352 | 3951 ± 210 | 3234 ± 272 |
| | 128 | CFU-S/femur | 4005 ± 342 | 4283 ± 173 | 3856 ± 172 | 3184 ± 180 |
| | 256 | | 3110 ± 314 | 3401 ± 88 | 4307 ± 157 | 3724 ± 198 |
| | 0 | | 24.3 ± 1.2 | 21.6 ± 0.6 | 27.9 ± 0.7 | 20.2 ± 2.0 |
| | 128 | Cells/femur | 23.5 ± 0.3 | 18.6 ± 1.2 | 27.0 ± 1.0 | 21.2 ± 2.3 |
| | 256 | (× $10^{-6}$) | 25.7 ± 1.3 | 22.8 ± 2.4 | 27.5 ± 0.8 | 16.2 ± 1.1 |
| | 0 | | 570 ± 72 | 629 ± 35 | 560 ± 53 | 567 ± 80 |
| 19 weeks | 128 | CFU-F/femur | 362 ± 50 | 356 ± 32 | 547 ± 51 | 67 ± 15 |
| | 256 | | 514 ± 55 | 415 ± 42 | 261 ± 92 | 1288 ± 76 |
| | 0 | | 3421 ± 172 | 5043 ± 258 | 5280 ± 131 | 2780 ± 81 |
| | 128 | CFU-S/femur | 3362 ± 256 | 5017 ± 289 | 3989 ± 183 | 3118 ± 148 |
| | 256 | | 3569 ± 259 | 5838 ± 529 | 3663 ± 203 | 3145 ± 255 |

Each result is the mean of 5 mice assayed individually ( ± s.e.). Total mice assayed = 60 at each level of $^{239}$Pu injection. ND = 12 week data for CFU-F at PICR were lost due to technical problems.

**Table 3** Bone marrow assays – averages of data for both centres and all ages of animals

| Assay | BDF1 | CBA/H | $^{239}$Pu (Bq $g^{-1}$) |
|---|---|---|---|
| Cells/femur | 22.7 ± 0.7 | 22.7 ± 0.7 | 0 |
| (× $10^{-6}$) | 21.8 ± 0.6 | 21.2 ± 0.8 | 128 |
| | 21.4 ± 0.7 | 22.0 ± 0.7 | 256 |
| CFU-F/femur | 454 ± 41 | 545 ± 50 | 0 |
| | 352 ± 26 | 410 ± 47 | 128 |
| | 395 ± 28 | 593 ± 80 | 256 |
| CFU-S/femur | 3992 ± 162 | 3653 ± 182 | 0 |
| | 4168 ± 143 | 3363 ± 140 | 128 |
| | 3969 ± 213 | 3711 ± 110 | 256 |

Each result is the mean of 30 mice, assayed individually (± s.e) at each centre, for the three age groups combined.

Metaphase preparations of marrow and spleen cells were made by standard procedures and stained with 5% Giemsa. One cytogeneticist at each of the two centres was responsible for chromosome analysis. Usually 50 (occasionally 100) cells were scored from marrow and spleen preparations from each animal. All scoring was done on coded and randomized slides.

Marrow preparations were made from 42-, 84- and 126-day-old animals at MRC but only from 42- and 84-day-old animals at PICR (except for 7-day cultures from BDF1 mice at 126 days). Spleen preparations were made from 42- and 84-day-old animals at both centres.

### RESULTS

### Fertility

Although at the higher dose of $^{239}$Pu, the mice were slower to mate and for the BDF1 a second batch of pairings had to be introduced in order to obtain sufficient litters, there were no significant differences in the size of those litters born. CBA/H litter sizes for the control, 128 and 256 Bq $g^{-1}$ $^{239}$Pu groups were respectively 6.7, 7.3 and 6.1. BDF1 litters similarly averaged 6.4, 6.9 and 6.3 respectively (Table 1).

### Bone marrow

Bone marrow cellularity, CFU-S and CFU-F content were assayed in five mice individually at 6–8, 12 and 19 weeks of age for each of the two resultant sets of offspring. Average values for each group of five mice are shown in Table 2 and are further condensed to give average values for the two mouse strains over the whole experimental period in Table 3. Preconceptional paternal $^{239}$Pu appeared to have little effect on these average group values.

© Cancer Research Campaign 1998

**Figure 1** Frequency distributions of the numbers of cells per femur among the offspring of carrier treated fathers (**A**) and of fathers injected with 128 Bq $g^{-1}$ (**B**) or 256 Bq $g^{-1}$ (**C**) $^{239}$Pu. The two separate shades, plotted as a stacked bar histogram, indicate the data obtained for 20 individual assays at PICR (■) and 30 individual assays at MRC (▒) on both strains of mouse for each set of observations (total number of assays = 150)

**Figure 2** Frequency distributions of the numbers of CFU-F per femur among the offspring of carrier treated fathers (**A**) and of fathers injected with 128 Bq $g^{-1}$ (**B**) or 256 Bq $g^{-1}$ (**C**) $^{239}$Pu. Bars show the data obtained for 30 individual assays at PICR (■) and MRC (▒) and for each set of observations (total number of assays = 180)

A two-sided analysis of variances on the raw control data, comparing each strain of mouse assayed at PICR vs MRC and comparing CBA/H vs BDF1 mice at each centre, was carried out. Generally lower measurements for femoral cellularity in CBA/H mice at MRC were significant at the 1% level. This difference was less evident for BDF1 mice ($P = 0.05$). However, at neither centre was there a significant overall difference between the two strains (Table 3). These cell counts are integral in CFU-S and CFU-F calculations and the differences are therefore reflected in lower CFU-S values ($P = 0.01$), where CBA/H measurements at MRC were involved in the comparisons. It led also to a marginal difference in CBA/H–CFU-F values between PICR and MRC ($P = 0.05$). No other comparison produced significant differences between strains or centres.

To compare the overall effects of PPI, and to compensate for minor variations resulting from assays at different times, different venues and different mice, average values for all the individual control mice were calculated. Values obtained for cellularity, CFU-S and CFU-F were respectively $22.7 \times 10^6$, 3800 and 480 per femur. Then, based on the mean control value for each subset of observations (e.g. 12 week BDF1 mice at PICR etc.), the values for each individual mouse were normalized against the appropriate overall average value. These normalized data were plotted as frequency histograms (Figures 1–3). Figure 1A shows the femoral cellularities in control offspring to be normally distributed, a mean value of $22.7 \times 10^6 \pm$ a standard deviation (s.d.) of $4.1 \times 10^6$ incorporating 70% of the total observations. Parity of the data between the two centres is shown in Figure 1A, where the separate data sets for the control animals are identified and seen to contribute approximately equally to the distribution. A two-sided analysis of variance on the standard deviations showed no significant difference between the two centres. This normal distribution was maintained in the offspring of plutonium-injected fathers – about 80% of the observations lying within the control standard deviation, though a slight broadening and left-shift of the peak frequencies may be perceived (Figure 1B and 1C). Although an analysis of variance showed no significant change in the mean value, the standard deviations of the groups became significantly different at 256 Bq $g^{-1}$ $^{239}$Pu compared with control ($P = 0.02$ at PICR and $P = 0.05$ at MRC).

Figure 2A shows the CFU-F for normal offspring also to be normally distributed, a mean value of $480 \pm$ a standard deviation (s.d.) of 156, incorporating 76% of the individual assays, and there was no significant difference between the distributions for PICR

 © Cancer Research Campaign 1998

**Figure 3** Frequency distributions of the numbers of CFU-S per femur among the offspring of carrier treated fathers (**A**) and of fathers injected with 128 Bq g$^{-1}$ (**B**) or 256 Bq g$^{-1}$ (**C**) $^{239}$Pu. Bars show data obtained for 30 individual assays at PICR (■) and MRC (▒) and for each set of observations (total number of assays = 180)

compared with MRC. However, 128 Bq g$^{-1}$ $^{239}$Pu in the father caused the distribution to spread more uniformly over a greater range of CFU-F per femur (Figure 2B), 58% then being outside the normal range of 1 s.d. At 256 Bq g$^{-1}$ the distribution split into two distinct groups of low and high numbers of CFU-F per femur, though it should be noted that all the high values were reported for one centre – MRC (Figure 2C) – the difference with PICR being significant at $P < 0.01$. Of these, 70% were outside the normal range of 1 s.d. Overall mean group values were similar, but analysis of variance on the standard deviations showed significant differences in the distributions at 128 Bq g$^{-1}$ ($P < 0.01$) and 256 Bq g$^{-1}$ ($P < 0.001$). A similar trend was evident for the femoral CFU-S content. Of the normal mice, 82% contained CFU-S within 1 s.d. of 546 on a mean value of 3800 per femur, with no significant difference between results from PICR and MRC (Figure 3A). Increasing amounts of $^{239}$Pu to the father (Figure 3B and 3C) caused the distribution increasingly to flatten and extend such that 47% and 52% lay outside the normal range of 1 s.d. respectively. In this case, results from PICR and MRC for the PPI groups were not significantly different. Neither was the 128 Bq g$^{-1}$ distribution different from the controls. At 256 Bq g$^{-1}$, however, the increased spread in the distribution (Figure 3C) was different ($P = 0.01$).

**Figure 4** Aberration yields relative to untreated controls. The two high values at 128 Bq g$^{-1}$ reflect the low control frequencies in these groups of animals

### Cytogenetics

Chromosome aberration data are presented in Table 4, subdivided into three main datasets: spleen, 2-day bone marrow cultures and 7-day bone marrow cultures. Aberrations in all three groups were mainly gaps and breaks (predominantly chromatid-type) with 10% exchanges or less. The results can be summarized as follows:

(1) At both centres there was significant evidence of differences between aberration yields in replicate mice. This was true within each of the three datasets and is allowed for in subsequent statistical tests.
(2) Within each dataset there were some significant differences in aberration yield between the two centres, but these differences were not consistent. For example, for the CBA/H 6-week group, considering spleen cells at all doses (0, 128 and 256 Bq g$^{-1}$), yields at PICR were significantly lower than at MRC ($P = 0.03$). In contrast, in the BDF1 6–8 week group, yields in spleen cells were significantly higher at PICR than at MRC ($P = 0.01$).
(3) For bone marrow cells, there was no significant difference between results in the two mouse strains. For spleen cells, there were differences within some data subsets: for example, at PICR, yields in BDF1 mice (all doses and ages) were, overall, higher than in CBA mice ($P = 0.022$).
(4) For spleen cells, there was no significant difference in yields between animals sampled at different ages. However, for marrow cells there was considerable heterogeneity between data subsets with no consistent pattern.

**Table 4** Chromosome aberration data including gaps (achromatic lesions)

| Cell origin | Centre | Strain | Age (weeks) | Aberration yield (%) at dose (Bq $g^{-1}$) | | | Ratio of yields | |
|---|---|---|---|---|---|---|---|---|
| | | | | 0 | 128 | 256 | 128:0 | 256:0 |
| Spleen | PICR | CBA/H | 6 | 6.0 ± 1.8 | 4.4 ± 1.6 | 5.2 ± 1.7 | 0.73 ± 0.35 | 0.87 ± 0.39 |
| | | | 12 | 9.6 ± 2.3 | 9.6 ± 2.3 | 5.2 ± 1.7 | 1.00 ± 0.34 | 0.54 ± 0.22 |
| | | BDF1 | 6 | 10.7 ± 3.2 | 12.0 ± 3.4 | 8.7 ± 2.9 | 1.12 ± 0.46 | 0.81 ± 0.36 |
| | | | 12 | 8.0 ± 2.8 | 12.7 ± 3.5 | 8.0 ± 2.8 | 1.58 ± 0.70 | 1.00 ± 0.49 |
| | MRC | CBA/H | 8 | 6.0 ± 2.1 | 10.4 ± 2.4 | 9.6 ± 2.3 | 1.73 ± 0.72 | 1.60 ± 0.67 |
| | | | 12 | 2.0 ± 1.1 | 5.3 ± 2.2 | 4.0 ± 1.8 | 2.67 ± 1.81 | 1.99 ± 0.14 |
| | | BDF1 | 8 | 4.0 ± 1.5 | 6.0 ± 1.8 | 6.0 ± 1.8 | 1.5 ± 0.73 | 1.50 ± 0.73 |
| | | | 12 | 7.6 ± 2.1 | 5.6 ± 1.8 | 4.8 ± 1.7 | 0.74 ± 0.31 | 0.63 ± 0.28 |
| | | | | | | | Overall ratios* | |
| | | | | | | | 1.19 ± 0.18 | 0.95 ± 0.15 |
| Marrow (2 day) | PICR | CBA/H | 6 | 10.8 ± 2.5 | 11.6 ± 2.6 | 11.2 ± 2.6 | 1.07 ± 0.35 | 1.04 ± 0.34 |
| | | | 12 | 11.6 ± 2.6 | 6.8 ± 2.0 | 7.6 ± 2.1 | 0.59 ± 0.22 | 0.66 ± 0.23 |
| | | BDF1 | 6 | 9.2 ± 2.3 | 9.2 ± 2.3 | 12.4 ± 2.7 | 1.00 ± 0.36 | 1.35 ± 0.45 |
| | | | 12 | 8.0 ± 2.2 | 7.2 ± 2.1 | 8.8 ± 2.3 | 0.90 ± 0.36 | 1.10 ± 0.41 |
| | MRC | CBA/H | 8 | 14.8 ± 2.9 | 20.0 ± 3.4 | 22.4 ± 3.6 | 1.35 ± 0.35 | 1.51 ± 0.39 |
| | | | 12 | 4.9 ± 3.0 | 11.0 ± 2.8 | 13.2 ± 2.8 | 2.22 ± 1.45 | 2.67 ± 1.71 |
| | | | 19 | 16.4 ± 3.1 | 28.8 ± 4.1 | 29.2 ± 4.1 | 1.76 ± 0.42 | 1.78 ± 0.42 |
| | | BDF1 | 8 | 11.3 ± 3.3 | 22.0 ± 3.6 | 24.4 ± 3.8 | 1.94 ± 0.65 | 2.15 ± 0.71 |
| | | | 12 | 10.0 ± 2.4 | 15.2 ± 3.0 | 15.2 ± 3.0 | 1.52 ± 0.47 | 1.52 ± 0.47 |
| | | | 19 | 22.0 ± 3.6 | 24.8 ± 3.8 | 38.0 ± 4.7 | 1.13 ± 0.25 | 1.73 ± 0.35 |
| | | | | | | | Overall ratios* | |
| | | | | | | | 1.27 ± 0.12 | 1.47 ± 0.14 |
| Marrow (7 day) | PICR | CBA/H | 6 | 7.2 ± 1.6 | 8.7 ± 2.0 | 17.0 ± 2.5 | 1.22 ± 0.39 | 2.36 ± 0.63 |
| | | | 12 | 12.0 ± 2.9 | 11.6 ± 2.9 | 10.0 ± 2.7 | 0.97 ± 0.34 | 0.83 ± 0.30 |
| | | BDF1 | 6 | 6.4 ± 2.1 | 8.0 ± 2.4 | 12.8 ± 3.0 | 1.25 ± 0.56 | 2.00 ± 0.82 |
| | | | 12 | 18.8 ± 3.7 | 16.8 ± 3.5 | 14.4 ± 3.2 | 0.89 ± 0.25 | 0.77 ± 0.23 |
| | | | 19 | 18.0 ± 3.6 | 23.3 ± 4.1 | 12.8 ± 3.0 | 1.29 ± 0.34 | 0.71 ± 0.22 |
| | MRC | CBA/H | 8 | 6.0 ± 2.1 | 8.0 ± 2.4 | 16.4 ± 3.4 | 1.33 ± 0.61 | 2.73 ± 1.10 |
| | | | 12 | 0.8 ± 0.8 | 8.7 ± 3.2 | – | 10.83 ± 10.98 | – |
| | | | 19 | 11.6 ± 2.9 | 18.4 ± 3.6 | 16.4 ± 3.4 | 1.59 ± 0.50 | 1.41 ± 0.46 |
| | | BDF1 | 8 | 12.7 ± 3.9 | 14.0 ± 3.2 | 13.2 ± 3.1 | 1.11 ± 0.42 | 1.04 ± 0.40 |
| | | | 12 | 2.0 ± 1.3 | 11.3 ± 2.9 | 3.6 ± 1.6 | 5.68 ± 4.06 | 1.81 ± 1.45 |
| | | | 19 | 6.4 ± 2.1 | 12.7 ± 3.9 | – | 1.98 ± 0.90 | – |
| | | | | | | | Overall ratios* | |
| | | | | | | | 1.37 ± 0.23 | 1.38 ± 0.24 |

*Assuming that aberration yields at doses 0, 128 and 256 Bq $g^{-1}$ are in the ratio of $1{:}y_1{:}y_2$, each individual line of the Table yields an estimate of this ratio. The overall ratios quoted are the maximum likelihood estimates of $y_1$ and $y_2$ provided by all relevant lines of the table, after first testing for heterogeneity among the individual ratios. Individual ratios were satisfactorily homogeneous in the cases of spleen and 2 day bone marrow, but were significantly heterogeneous in the case of 7 day bone marrow. Standard errors in the latter case have been increased by the appropriate heterogeneity factor.

(5) In spite of the heterogeneity of results under 1–4, and taking this into account in the statistical analysis, there was evidence of a significant increase in aberration yields with dose in marrow cells. Overall mean ratios of yields for 2-day marrow samples were 1.00:1.27:1.47 at 0, 128 and 256 Bq $g^{-1}$ respectively (Table 4 and Figure 4), the yields showing a good fit to the linear model (yield = $\alpha + \beta D$) with a slope ($\beta$) of $(1.50 \pm 0.35) \times 10^{-3}$.

For the 7-day marrow samples, the ratios of yields at 0, 128 and 256 Bq $g^{-1}$ were 1.00:1.37:1.38, with evidence of saturation. Nevertheless, the data were an adequate fit to the linear model, with $\beta = (1.17 \pm 0.67) \times 10^{-3}$. For spleen cells there was no significant evidence of an effect of dose on aberration yields (Tables 4 and 5).

Dose–response relationships were similar whether or not gaps were included in the analysis of chromosome aberration data (Table 5).

### Lymphoma/leukaemia following administration of MNU

Following a single injection of MNU at 10 weeks of age, BDF1 mice were observed at regular intervals for the first 2 months, during which time none showed adverse symptoms, and thereafter daily. The first symptoms of disability appeared at 61 days in an animal whose father received 128 Bq $g^{-1}$ $^{239}Pu$ (Figure 5). Eight days later, it became necessary to sacrifice the first mouse from the 256 Bq $g^{-1}$ group. Only at 28 days later did the first of the control group show signs of disease. This was significantly later than for the 128 Bq $g^{-1}$ group ($P = 0.03$), but not for the higher dose group. Subsequent animals were sacrificed as symptoms of deteriorating health appeared. In the control group, thymic lymphomas and leukaemias accumulated at a steady rate, exactly in agreement with an earlier report (Schofield & Dexter, 1974), and by 185 days 50% had succumbed (Figure 5 and Table 6). Animals in the plutonium groups had to be sacrificed at a faster rate than those in the control group, 50% incidences arising by 161 days ($P = 0.002$,

© *Cancer Research Campaign 1998*

regression analysis of the log-probit slopes) and by 125 days ($P \ll 0.001$) in the 256 and 128 Bq $g^{-1}$ groups respectively. It is notable that the minimum latent period was shortest and the rate of incidence of disease was greatest in the offspring of fathers who had received the lower amount of plutonium. The disease/incidence data are summarized in Table 6.

In general, two types of malignancy were expected and obtained. In controls (and as previously reported, Dexter et al, 1974), the primary problem was development of thymic lymphoma, characterized by a grossly enlarged thymus, but no other tissue abnormality. Over a period of 4–5 months, about two-thirds of the mice sacrificed bore thymic lymphomas only, while, accruing more slowly, only one-third developed other malignancies, primarily leukaemias involving bone marrow, spleen, lymph nodes and liver (Figure 6). The rates of accumulation of each type of malignancy were not statistically different (variance analysis on the individual times of incidence), but the shortest latent period was less for the lymphomas than for the others ($P < 0.01$).

This pattern of disease was reversed in the two groups whose fathers received plutonium. About 70% of the mice sacrificed were leukaemic, while only 30% had disease limited to the thymus (Figure 6 and Table 6). Both groups of disease developed with a shortened minimum latent period, which in all cases was highly significant ($P \ll 0.001$). The rate of onset of thymic lymphoma – but not leukaemia – was significantly lower than that in the controls ($P \ll 0.001$).

**Figure 5** Cumulative incidence of thymic lymphomas and leukaemias with time following a single injection of 50 mg $kg^{-1}$ of MNU. The data are plotted on log-probit axes. Each symbol represents one case (or group of cases when multiple cases arose on the same day) and indicates offspring of carrier treated fathers (●), and fathers injected with 128 Bq $g^{-1}$ (▲) or 256 Bq $g^{-1}$ (■) $^{239}$Pu

### DISCUSSION

### Effect of PPI on lymphoma/leukaemia induction by MNU

In this study we have sought to investigate the possibility that PPI, in the form of an intravenous injection of $^{239}$Pu, 12 weeks before mating with a normal mouse, results in transmitted defects such that the offspring are more susceptible to the lymphogenic/leukaemogenic effects of a chemical carcinogen – MNU – encountered post-natally. Doses of $^{239}$Pu were chosen such that no direct effect on fertility would be induced. Previously, 128 Bq $g^{-1}$ $^{239}$Pu was shown to have no effect on fertility or litter size, despite a sperm count reduced to 15% of control (Searle et al, 1976). In this study, the higher dose of 256 Bq $g^{-1}$ reduced the frequency of pregnancies somewhat, but as with 128 Bq $g^{-1}$ it had no effect on litter size (Table 1).

In a large proportion of adult mice, MNU induces primarily thymic lymphomas, characterized by a grossly enlarged thymus but no other overt change and, developing later and more slowly, myeloid leukaemias involving the bone marrow and spleen (Dexter et al, 1974). These results were reproduced exactly in our control group (Figures 5 and 6). PPI modified this pattern of development such that the minimum latent periods for both categories of disease were shortened, and the more dominant development of disease involving the bone marrow and spleen (the leukaemias) appeared to suppress the rate of development of thymic lymphoma. Thus, all three parameters of MNU-induced malignancy were significantly modified: there was a reduced latency period, an increased rate of incidence and level of malignancy and

**Table 5** Statistical analyses of dose–response data for chromosome aberrations

| Dataset[1] | Ratios of yields | | Fit to linear model $Y = \alpha + \beta D$ | | | Slope ($\times10^{-3}$) | Significance of slope | | |
|---|---|---|---|---|---|---|---|---|---|
| | 128:0 | 256:0 | $F$ | df. | $P$ | | $F$ | df. | $P$ |
| Spleen + gaps[2] | 1.19 ± 0.18 | 0.95 ± 0.15 | NA[4] | | | NA | NA | | |
| Spleen – gaps[3] | 1.09 ± 0.20 | 0.76 ± 0.15 | NA | | | NA | NA | | |
| Marrow (2 day) + gaps | 1.27 ± 0.12 | 1.48 ± 0.14 | 0.878 | 19, 117 | 0.61 | 1.50 ± 0.35 | 18.6 | 1,117 | <0.001 |
| Marrow (2 day) – gaps | 1.36 ± 0.16 | 1.61 ± 0.18 | 0.912 | 19, 117 | 0.57 | 1.80 ± 0.42 | 17.99 | 1,117 | <0.001 |
| Marrow (7 day) + gaps | 1.37 ± 0.23 | 1.38 ± 0.24 | 1.24 | 1,18 | 0.28 | 1.17 ± 0.67 | 3.10 | 1,19 | 0.094 |
| Marrow (7 day) – gaps | 1.43 ± 0.26 | 1.44 ± 0.27 | 1.40 | 1,18 | 0.25 | 1.36 ± 0.71 | 3.69 | 1,19 | 0.070 |

[1]Combined data for both centres, both mouse strains and all sampling times; [2]ratios not significantly different from 1.00 [$F$(2,81) = 1.17; $P$ = 0.32]; [3]ratios not significantly different from 1.00 [$F$(2,81) = 1.67; $P$ = 0.19]; [4]not applicable.

© Cancer Research Campaign 1998

**Figure 6** Relative cumulative incidences of thymic lymphomas (●) and leukaemias (▲) in offspring of carrier-treated fathers (**A**) and fathers injected with 128 Bq $g^{-1}$ (**B**) or 256 Bq $g^{-1}$ (**C**) $^{239}Pu$. The first case (shown in **B**) developed 60 days after a single injection of MNU and is designated time-zero on the abscissae a switch from the predominant thymic lymphoma in the controls to predominance of leukaemias in the offspring of plutonium-treated fathers.

These studies therefore corroborate Nomura's (1983) suggestion that offspring of PPI may have increased susceptibility to tumour induction. Two other studies have indicated the potential of preconceptional paternal irradiation to increase the sensitivity of their offspring to a secondary carcinogenic insult. Vorobtsova and her colleagues reported the incidence of urethane-induced lung adenomas (Verobtsova & Kitaev, 1988) and of skin papillomas induced by 12-O-tetracanoylphorbol-13-acetate (Verobtsova et al. 1993) in mice following a paternal dose of X-rays shortly before mating. The defect in both cases was found to be transmitted through at least two generations.

**Table 6** Summary of offspring lymphoma/leukaemias following injection of MNU

| | Control | 128 Bq $g^{-1}$ $^{239}Pu$ | 256 Bq $g^{-1}$ $^{239}Pu$ |
|---|---|---|---|
| Number injected with MNU | 55 | 62 | 41 |
| Time to first case (days) | 89 | 61 | 69 |
| Time to 50% incidence of malignancy (days) | 185 | 125$^{a}$ | 161$^{b}$ |
| Rate of incidence (% per day to 50%) | 0.510 | 0.725 | 0.562 |
| Minimum latent period (days) | 89 | 61$^{c}$ | 69$^{c}$ |
| Lymphomas* (%) | 62 | 35 | 35 |
| Leukaemias** (%) | 38 | 65 | 65 |

*Thymic involvement only (by gross pathology). **Bone marrow, spleen (with or without thymic) involvement. Over 95% of the animals sacrificed demonstrated lymphohaemopoietic involvement. Significance compared with control: $^{a}P \ll 0.001$; $^{b}P = 0.002$; $^{c}P = 0.03$; $^{d}P = 0.05$.

In a separate large-scale experiment, there was no evidence of leukaemia induction by $^{239}Pu$ PPI alone (EB Humphreys and VA Stones, personal communication). It is clear, therefore, that $^{239}Pu$ injected into the male mouse before mating – a form of contamination which results in continuous irradiation to the spermatogenic process (Green et al. 1975) – can result in changes in the leukaemic susceptibility of the offspring to exposure to a secondary carcinogen/mutagen. It is interesting, however, that the greater change occurred in the lower $^{239}Pu$ dose group. A possible explanation was provided by Smith and Doll (1982), who suggested that radiation could cause leukaemic lesions at both high and low doses, but that at the higher doses cell sterilization would reduce their net development. It is possible that the higher paternal dose of $^{239}Pu$ similarly produced more spermatogenic death, in line with the lower fertility rate for this group, while the lower dose was responsible for more subtle damage which was subsequently transmitted to the offspring.

### Effect of PPI on haemopoiesis

While an altered response to MNU was evident following PPI, defined changes in haemopoiesis were less obvious. Minor differences in marrow cellularity of the control mice between the two centres, which were projected automatically into CFU-S and CFU-F measurements, were probably related to differences in husbandry practices: diet, handling, environment, etc. However, the distributions of individual mouse values for all parameters were normal and similar at both centres (Figures 1A, 2A and 3A). Peripheral blood patterns suggested that the PPI offspring were also haematologically normal and average bone marrow cellularities were normal, with a normal distribution among the individual mice. Progenitor cells, however, and in particular CFU-F, although showing no overall change, demonstrated considerably more inter-animal differences, and it was clear that PPI offspring and the controls must be considered as different populations. This corroborates our limited preliminary data on pooled groups of animals (Lord et al. 1995). In that study, apparent dose-related responses of the CFU-S and CFU-F to the lower amounts of plutonium used were replaced by variable responses at the higher dose levels that were subsequently used in this study. It is notable that, in the current experiments, a cohort of high CFU-F results was picked up

 © Cancer Research Campaign 1998

at MRC in the high-dose offspring. These mice are consistent with those in our preliminary group, which showed a 73% increase in 256 Bq $g^{-1}$ offspring. On this occasion, results at PICR were, on average, lower than the control values, but it must be emphasized that under the conditions of these experiments each animal must be considered an individual and it is probably fortuitous that the overall average value is similar to that of the controls.

### Effects of PPI on cytogenetics of haemopoietic tissue

As in the preliminary experiment, therefore, PPI results in random perturbation of haemopoietic tissue and in at least one component of its regulatory microenvironment. It is possibly for the same reasons that there was a parallel tendency for chromosome damage to be higher in PPI offspring. Alternatively, there is now abundant evidence of radiation-induced destabilization of the genome that can be transmitted over many generations of somatic cells and manifested as the delayed appearance of cell death, chromosome damage and gene mutations (reviewed by Morgan et al, 1996). Our results suggest that such destabilization can also be transmitted through the germ line and expressed as elevated levels of chromosome aberrations in bone marrow cells of PPI offspring. Similar observations in rats have been reported by Vorobtsova (1989, 1995) and also in humans – on lymphocytes from the children of workers involved in the clean-up after the Chernobyl nuclear accident and from children born to parents who had received radiotherapy for Hodgkin's disease (Vorobtsova and Voreb'eva, 1992; Vorobtsova, 1995; Vorobtsova et al, 1995). Genomic instability was detected as elevated levels of 'spontaneous' chromosome damage or enhanced chromosomal radiosensitivity of lymphocytes of the children. Germline-transmitted instability, but with different end points, has also been described by Lunning et al (1976) for dominant lethality in mice and by Luke et al (1997) for mutations in mouse transgene. In our study, the chromosome instability seen in the bone marrow of PPI offspring was not seen in spleen cells (Table 3). It is possible that this reflects cell type differences, as is seen in the expression of the instability phenotype in somatic cells (Kadhim et al, 1995; Morgan et al, 1996).

### Haemopoiesis and leukaemogenesis

In view of these perturbations in haemopoiesis, it is tempting to speculate on their link to the changed pattern of lympho-haemopoietic malignancy. The normal bone marrow cellularity and committed progenitor cell levels (Lord et al, 1995) suggest compensatory changes in proliferative activity throughout the self-renewal, commitment and maturation processes when the multipotent progenitor (stem cell) population is not normal. When there is a reduction in CFU-S, proliferation is triggered (Schofield & Lord, 1984) and is seen, for example, when injected $^{239}$Pu reduces the CFU-S population (Lord et al, 1991; Mason et al, 1992). Complementary to changes in haemopoietic function is potential damage to the stromal microenvironment for haemopoietic tissue. Studies of renal bone capsule-forming capacity in marrow from fetal (Lord et al, 1992; Mason et al, 1992) and adult (Lord et al, 1991) mice injected with $^{239}$Pu, or of stromal layer function in long-term bone marrow cultures of $^{241}$Am-treated mice (van den Heuvel, 1990), have indicated significant involvement of damage to stromal cells. In addition, these studies, which assess the potential of the whole haemopoietic microenvironment complex, have been corroborated with CFU-F measurements in γ-irradiated mice (Yang et al, 1995). CFU-F, together with other components of the stromal microenvironment, provide the appropriate balance of growth-stimulating and -inhibiting factors. Furthermore, damage to this system is probably a major contributor to changes in stem cell proliferative activity. Chromosomal damage to the stem cells, which was seen to increase in the PPI offspring, may well be secondary to induced proliferation. Normally, the stem cell populations spend most of their time in a non-proliferative state – a time for the 'genetic housekeeping' necessary to preserve the integrity of the genome. Hyperproliferative activity reduces the time spent in this state and so increases the risk of subsequent undesirable mutations, the expression of which may be increased or accelerated by encountering a secondary carcinogenic insult. We did not, in these experiments, measure the proliferative activity of CFU-S in the individual mice, but it might be expected that in situations of low CFU-S number and/or low CFU-F number, they will be in this vulnerable, highly proliferative state. The increasing rate of chromosome aberrations may well argue against a simple acceleration of the development of leukaemias that would arise anyway with MNU, but in either case it clearly takes the secondary exposure to the carcinogen/mutagen to expose the PPI damage which on its own is insufficient to induce leukaemias in mice.

### CONCLUSION

From its deliberations, COMARE (1996) concluded that there was no convincing evidence of leukaemia or lymphoma from human studies involving paternal irradiation and found inconsistencies in the animal experiments that may in part have been due to a lack of concurrent controls, whose periodic or cyclic variation in tumour incidence may have been out of phase with that in the treated animals (Cattenach et al, 1995). Concurrent controls were incorporated in these present experiments. While there are no grounds for suggesting that these results explain the Seascale phenomenon, we believe this current report is the first to describe the enhanced induction of lympho-haematological disorders following preconceptional paternal irradiation. Together with the urethane and phorbol acetate studies, these observations present evidence that offspring of an irradiated parent (male) may be at increased risk when exposed to secondary carcinogenic noxae. This may be the result of perturbed haemopoiesis – in the case of these present experiments – or possibly by a mechanism involving the activation of endogenous retroviral elements by radiation (Erfle et al, 1986; Mitreiter et al, 1994) which, if genetically transmitted, may render the next generation more sensitive to such secondary promoters. There is, however, no evidence for such a mechanism in these experiments.

### Summary

(1) PPI, resulting from $^{239}$Pu injection, in mice reduces the minimum latent period and increases the rates of incidence and profile of lympho-haemopoietic malignancies induced by subsequent exposure to the chemical carcinogen–mutagen – methyl-nitroso-urea.

(2) Haematologically, the production of functional blood cells is normal in PPI mice. At the level of the individual mouse, however, the kinetics of haemopoiesis are modified such that normal levels of production are maintained from abnormal

© Cancer Research Campaign 1998 

levels (low or high) of pluripotent progenitor cells and/or the stromal cell populations that regulate the process. Chromosomal aberrations, which must be the result of transmitted spermatogenic radiation damage, also increase in PPI mice.

(3) These observations suggest a potential mechanism whereby PPI may render specific individuals at greater risk if they are subsequently exposed to a further carcinogenic insult. It is possible that human populations, offspring of fathers similarly exposed, albeit at considerably lower dose levels, could also carry such an increased susceptibility to a secondary insult.

### ACKNOWLEDGEMENTS

This work was supported by the UK Cancer Research Campaign and the Medical Research Council together with a grant from the United Kingdom Coordinating Committee on Cancer Research.

### REFERENCES

Cattenach BM, Patrick G, Papworth D, Goodhead DT, Hacker T, Cobb L and Whitehill E (1995) Investigation of lung tumour induction in BALB/c mice following paternal X-irradiation. *Int J Radiat Biol* **67**: 606–616

Comare (1996) Possible effects of paternal preconception irradiation in cancer. Committee on Medical Aspects of Radiation in the Environment. 4th report. pp. 82–96. Department of Health: London

Dexter TM, Schofield R, Lajtha LG and Moore M (1974) Studies on the mechanisms of chemical leukaemogenesis. *Br J Cancer* **30**: 325–331

Doll R, Evans HJ and Darby SC (1994) Paternal exposure not to blame. *Nature* **367**: 678–680

Erfle V, Schmidt J, Strauss GP, Hehlmann R and Luz A (1986) Activation and biological properties of endogenous viruses in radiation osteosarcomagenesis. *Leuk Res* **10**: 905–913

Friedenstein AJ, Chailakjan RK and Lalykina KS (1970) The development of fibroblast colonies in monolayer cultures of guinea-pig bone marrow and spleen cells. *Cell Tissue Kinet* **3**: 393–403

Friedenstein AJ, Luria E and Molineux G (1993) Assays of the haemopoietic microenvironment. In *Haemopoiesis: A Practical Approach*. Testa NG and Molineux G (eds). pp. 189–199. IRL Press: Oxford

Gardner MJ, Snee MP, Hall AJ, Powell CA, Downes S and Terrell JD (1990) Results of case–control study of leukaemic and lymphoma among young people near Sellafield nuclear plant in West Cumbria. *Br Med J* **300**: 423–429

Green D, Howells GR, Humphreys ER and Vennart J (1975) Localisation of plutonium in mouse testes. *Nature* **255**: 77

Humphreys EB, Loutit JF and Stones VA (1987) The induction by $^{239}$Pu of myeloid leukaemia and osteosarcoma in female CBA mice. *Int J Radiat Biol* **55**: 331–339

Kadhim MA, Lorimore SA, Townsend KMS, Goodhead DT, Buckle VJ and Wright EG (1995) Radiation-induced genomic instability: delayed cytogenic aberations and apoptosis in primary human bone marrow cells. *Int J Radiat Biol* **67**: 287–293

Lord BI (1993) *In vivo* assays for multipotential and marrow repopulating cells. In: *Haemopoiesis: A Practical Approach*. Testa NG and Molineux G (eds). pp. 1–20. IRL Press: Oxford

Lord BI, Molineux G, Humphreys ER and Stones VA (1991) Long-term effects of plutonium-239 and radium-224 on the distribution and performance of pluripotent haemopoietic progenitor cells and their regulatory microenvironment. *Int J Radiat Biol* **59**: 211–227

Lord BI, Hendry JH, Keene JP, Hodgson BW, Xu CX, Rezvani M and Jordan TJ (1984) A comparison of low and high dose-rate radiation for recipient mice in spleen colony studies. *Cell Tissue Kinet* **17**: 323–334

Lord BI, Mason TM and Humphreys ER (1992) Age-dependent uptake and retention of $^{239}$Pu: its relationship to haemopoietic damage. *Radiat Protec Dosim* **41**: 163–167

Lord BI, Humphreys ER and Stones VA (1995) Preconceptual paternal plutonium-239 contamination: development of haemopoiesis in the offspring. In: *Health Effects of Internally Deposited Radionuclides*. van Kaick G, Karaoghou A and Kellerer AM (eds). pp. 341–345. World Scientific: Singapore

Luke GA, Riches AC and Bryant PE (1997) Genomic instability in haemopoietic cells of F1 generation mice of irradiated male parents. *Mutagenesis* **12**: 147–152

Luning KG, Frolen H and Nilsson A (1976) Genetic effects of Pu-239 salt injections in male mice. *Mutat Res* **34**: 539–542

Mabuchi K (1990) Malignant tumours during the first 2 decades of life in the offspring of atomic bomb survivors. *Am J Hum Genetic* **46**: 1041–1052

Mason TM, Lord BI, Molineux G and Humphreys ER (1992) Alpha-particle irradiation of haemopoietic tissue in pre- and post-natal mice. II. Effects of mid-term contamination with plutonium-239 *in utero*. *Int J Radiat Biol* **61**: 393–403

Mitreiter K, Schmidt J, Luz A, Atkinson MJ, Hofler H, Erfle V and Strauss GP (1994) Disruption of the murine p53 gene by insertion of an endogenous retrovirus-like element (Etn) in a cell line from radiation-induced osteosarcoma. *Virology* **200**: 837–841

Morgan WF, Day JP, Kaplan MI, McGhee EM and Limoli CL (1996) Genomic instability induced by ionizing radiation. *Radiat Res* **146**: 247–258

Nomura T (1975) Transmission of tumours and malformations to the next generation of mice subsequent to urethane treatment. *Cancer Res* **35**: 264–266

Nomura T (1982) Parental exposure to X-rays and chemicals induces heritable tumours and anomalies in mice. *Nature* **256**: 575–577

Nomura T (1983) X-ray induced germ line mutation leading to tumours: its manifestation in mice given urethane post-natally. *Mutat Res* **121**: 59–66

Nomura T (1990) Of mice and men? *Nature* **345**: 671

Parker L, Craft AW, Smith J, Dickenson H, Wakeford R, Binks K, McElvenny D, Scott L and Slovak A (1993) Geographical distribution of preconceptional radiation doses to fathers employed at the Sellafield nuclear installation, West Cumbria. *Br Med J* **307**: 966–971

Schofield R and Dexter TM (1974) Experimental leukaemogenesis. In: *Leukaemia and Aplastic Anaemia*. pp. 123–139. Il Pensiero Scientifico Editore: Rome

Schofield R and Lord BI (1984) $^{3}$H-thymidine suicide and the CFU-S population. *IRCS Med Sci* **12**: 779–780

Schofield R, Lord BI, Humphreys ER and Stones VA (1986) Effects of plutonium-239 on haemopoiesis. 1. Quantitative and qualitative changes in CFU-S in different regions of the mouse femur and vertebrae. *Int J Radiat Biol* **49**: 1021–1029

Searle AG, Beechey CV, Green D and Humphreys ER (1976) Cytogenetic effects of protracted exposure to alpha-particles from plutonium-239 and to gamma-rays from cobolt-60 compared in male mice. *Mutat Res* **41**: 297–310

Smith PG and Doll R (1982) Mortality among patients with ankylosing spondylitis after a single treatment course with X-rays. *Br Med J* **ii**: 449–460

Till JE and McCulloch EA (1961) A direct measurement of the radiosensitivity of normal mouse bone marrow cells. *Radiat Res* **14**: 213–222

Van den Heuvel RL (1990) Bone marrow from BALB/C mice radiocontaminated with $^{241}$Am in utero shows a deficiency in in vitro haemopoiesis. *Int J Radiat Biol* **57**: 103–115

Vorobtsova IE (1989) Increased cancer risk as a genetic effect of ionising radiation. In: *Perinatal and Multigenerational Carcinogenesis*. Napalkov NP, Rice JM, Tomatis L and Yamasaki H (eds). pp. 389–401. International Agency for Research on Cancer: Lyon

Vorobtsova IE (1995) Increased cancer risk and chromosomal instability in irradiated parents progeny (abstract). In: *Proceedings of the 25th Annual Meeting of the European Environmental Mutagenesis Society*. p. 52

Vorobtsova IE and Kitaev EM (1988) Urethane-induced lung adenomas in the first generation progeny of irradiated male mice. *Carcinogenesis* **9**: 1931–1934

Vorobtsova IE and Verob'eva MV (1992) The chromosomal radiosensitivity of children whose parents were exposed to antitumour radiochemotherapy (in Russian). *Biull Eksp Biol Med* **114**: 655–657

Vorobtsova IE, Aliyakparova LM and Anisimov VN (1993) Promotion of skin tumours by 12-O-tetradecanoylphorbol-13 acetate in two generations of descendants of male mice exposed to X-irradiation. *Mutat Res* **287**: 207–216

Vorobtsova IE, Voreb'eva MV, Bogomazova AN, Piukkenen A and Akkhangel'skaia TT (1995) The cytogenetic examination of children in the Saint Petersburg region who suffered as a result of the accident at the Chernobyl Atomic Electric Power Station. The frequency for unstable aberrations in the peripheral blood lymphocytes (in Russian). *Radiat Biol Radioecol* **35**: 630–635

Whitten WK (1958) Modification of the oestruns cycle of the mouse by exernal stimuli associated with the male. Changes in the oestruns cycle determined by vaginal smears. *J Endocrinol* **17**: 307–313

 *© Cancer Research Campaign 1998*

Yang FT, Lord BI and Hendry JH (1995) Gamma irradiation of the fetus damages the developing hemopoietic microenvironment rather than the hemopoietic progenitor cells. *Radiat Res* **141**: 309–313

Yoshimoto Y (1990) Cancer risk among children of atomic bomb survivors. A review of RERF epidemiological studies. *JAMA* **264**: 596–600

Yoshimoto Y, Neel JV, Schull WJ, Kato H, Soda M, Eto R and Mabuchi K (1990) Malignant tumours during the first 2 decades of life in the offspring of atomic bomb survivors. *Am J Hum Genet* **46**: 1041–1052

© Cancer Research Campaign 1998